# Prevalence of Diabetes Mellitus and Clinical Differences in Patients with Severe Osteoporosis and Fragility Fractures

**DOI:** 10.3390/jcm13092670

**Published:** 2024-05-02

**Authors:** Isabella Nardone, Rossella Antonelli, Simona Zaccaria, Sium Wolde Sellasie, Stefania Falcone, Chiara Pecchioli, Laura Giurato, Luigi Uccioli

**Affiliations:** 1 Division of Endocrinology and Diabetes, CTO Andrea Alesini Hospital, Department of Biomedicine and Prevention, University Tor Vergata, 00133 Rome, Italy; 2 PhD School of Applied Medical-Surgical Sciences, University of Rome Tor Vergata, 00133 Rome, Italy

**Keywords:** osteoporosis, diabetes mellitus, fragility fractures, bone mineral density (BMD), trabecular bone score (TBS), bone fragility

## Abstract

**Background: **Diabetes mellitus (DM) and osteoporosis are two of the most widespread metabolic diseases in the world. The aim of this study is to investigate the prevalence of DM among patients affected by osteoporosis and fragility fractures, and to search for differences in clinical characteristics. **Methods:** This is a single-center retrospective, case–controlled study. A total of 589 patients attending CTO Bone Unit between 2 January 2010 and 31 May 2023, due to osteoporosis and fragility fractures, were divided into two groups, according to the diagnosis of DM. The clinical and bone characteristics of patients were compared. **Results:** Prevalence of DM was 12.7%. Compared to patients without DM, the median age at the time of first fracture was similar: 72 years ± 13.5 interquartile range (IQR) vs. 71 years ± 12 IQR; prevalence of combination of vertebral and hip fractures was higher (*p* = 0.008), as well as prevalence of males (*p* = 0.016). Bone mineral density (BMD) at all sites was higher in DM group; trabecular bone score (TBS), instead, was significantly lower (*p* < 0.001). **Conclusions:** Patients with fragility fractures and DM more frequently show combination of major fractures with higher BMD levels. In these patients, TBS could be a better indicator of bone health than BMD and, therefore, might be used as a diagnostic tool in clinical practice.

## 1. Introduction

Osteoporosis is a metabolic disease of bone tissue, very common in the general population and even more common in the elderly [1]. This condition is characterized by a decrease in bone mineral density (BMD) and degradation of the tissue microarchitecture, with a progressive increase in the risk of fragility fractures [2]. The latter represent a serious health issue since they have heavy consequences from both a health and social point of view [3]. These consequences are even more severe when it comes to patients who have other serious comorbidities such as diabetes mellitus (DM). DM is another widespread metabolic disease [4], whose incidence is expected to grow quickly in the upcoming decades, due to the increase in average life expectancy and the epidemic diffusion of an unhealthy lifestyle [5]. In 2020, in Italy, the prevalence of type 2 DM was around 5.9% (3.5 million people). Prevalence tends to grow with age, reaching 21% in the population over 75 years of age [6]. Chronic complications of DM affect most body tissues [7], and bone is no exception. The existence of a complex crosstalk between glucose homeostasis and bone metabolism has been well described [8,9]. Both type 1 and type 2 DM, in fact, are recognized among the causes of secondary osteoporosis [10], leading to a reduced resistance to mechanical stress and to an increased risk of incurring fragility fractures in both sexes [11,12,13,14,15]. Chronic hyperglycemia, oxidative stress, and accumulation of advanced glycation end products (AGEs) are some of the most relevant mechanisms that cause abnormalities of the micro and macro architecture of the bone [16]. Proinflammatory cytokines and adipokines, exhibiting a direct toxic effect on osteoblasts differentiation, lead to the alteration of the physiological bone turnover which is slowed down [16]. Chronic complications of DM, as well as neuropathy, retinopathy, peripheral artery disease, poor balance, and sarcopenia, increase the risk of falls and subsequent fractures [17,18]. The risk is greater with a longer duration of DM, poor glycemic control, frequent hypoglycemic events, and certain DM therapies [13,19].

DM-related bone fragility is also characterized by the presence of a diagnostic paradox: despite the increased risk of fracture, patients with type 2 DM often show a normal or even increased BMD [20] at all sites evaluated by executing a dual energy X-ray absorptiometry (DXA) scan [20,21], the gold standard technique for the diagnosis of osteoporosis, so bone fragility may be often undiagnosed and not treated [22]. As previously reported, the trabecular microarchitecture appears to be altered in patients with DM; this parameter can be indirectly estimated in the lumbar spine using Trabecular Bone Score (TBS), a measure of grey level texture performed on lumbar images obtained by DXA [23,24]. TBS, estimating the 3D composition of the bone, allows the improvement, compared to the measurement of BMD alone, in the ability to predict fracture risk. In fact, a deterioration of TBS represents a risk factor for developing fragility fractures independently of BMD levels [25], and in a recent meta-analysis, a negative association between DM and TBS values was found [26]. Many studies have described the existence of relevant differences between BMD and TBS values in patients with and without DM. Do these differences remain clinically significant when we consider a selected population of patients who have already had fragility fractures? The purpose of the present study is to determine the prevalence and clinical characteristics of patients with DM in a population affected by fragility fractures and to evaluate whether BMD and TBS are significantly different between patients with and without DM even when only a population of patients who have already had fragility fractures is considered.

## 2. Materials and Methods

This is a single-center retrospective, case–controlled study.

### 2.1. Case Selection

We consulted the medical records of all consecutive patients affected by osteoporosis and fragility fractures attending the Bone Unit of the CTO Hospital of Rome between 2 January 2010 and 31 May 2023. Exclusion criteria were the presence of fractures due to major trauma, malignancy, and other causes not related to osteoporosis.

Patients were divided according to the presence of DM, for comparative analysis. The presence of DM was self-reported by patients during the first visit, confirmed and fully characterized by consulting the electronic medical record of the Diabetes Service of the CTO Hospital of Rome. The diagnosis of DM was confirmed by the finding of HbA1c greater than or equal to 6.5% (47 mmol/mol) at least twice, or a fasting blood glucose greater than or equal to 126 mg/dL on two different occasions, or HbA1c greater than or equal to 6.5% (47 mmol/mol) plus fasting blood glucose greater than or equal to 126 mg/dL at the same time, inside the electronic medical record. Informed written consent was obtained for the collection of clinical data.

### 2.2. Data Collection

For all patients, data on age, sex, body mass index (BMI—kg/m^2^), age at the time of the first fragility fracture, fracture site, smoking habit, excessive alcohol intake (more than 12 g of alcohol for females and more than 24 g of alcohol for males), self-reported diagnosis of rheumatoid arthritis (RA) and chronic obstructive pulmonary disease (COPD), long-term cortisone therapy (more than 5 mg/day of prednisone equivalents for more than 3 months), family history of osteoporosis (a close relative with a known low BMD condition or with one or more fragility fractures), BMD (g/cm^2^) assessed by DXA in the lumbar spine, femoral neck, and total hip, and TBS processed from DXA lumbar scan were collected from medical records. Fragility fractures were catalogued as vertebral fractures, hip fractures, and combination of vertebral and hip fractures and other non-vertebral or non-hip sites (clavicle, arm, elbow, pelvis, ankle, rib, tibia, foot, shoulder, knee). Fragility fractures were defined as spontaneous fractures or fractures that occurred after minor trauma or, in the case of asymptomatic vertebral collapses, that were diagnosed during radiological investigations.

For patients with DM, additional data were recorded: type of DM (type 1 or type 2); specific therapy (catalogued as nutritional therapy, oral hypoglycemic drugs, insulin therapy, combination of insulin therapy and oral hypoglycemic drugs). Information on DM chronic complications was made available by the systematic screening of chronic complications recorded in the electronic medical record of the Diabetes Service and were reported as follows: peripheral neuropathy, retinopathy, nephropathy, coronary heart disease, carotid artery atheromasia, and peripheral arterial disease. In addition, illness duration (years) and glycated hemoglobin (HbA1c) (%—mmol/mol) at the time of the first visit at the Bone Unit were collected.

### 2.3. BMD Assessment

BMD was assessed by skilled operators in our center by DXA [21] in the lumbar spine, femoral neck, and total hip, using an Hologic Discovery densitometer. In the lumbar scan, vertebrae that presented artefacts from arthrosis or vertebral collapse were excluded from the BMD calculation. Daily phantom scans were performed for quality control prior to clinical activity. Osteoporosis was diagnosed in presence of a T-score value at or below −2.5 SD, or in presence of fragility fractures if T-score values were higher than −2.5 SD. TBS iNsight Imaging Software (version 3.0.2.0, Medimaps Group, Geneva, Switzerland) was used to elaborate TBS from DXA scan of the lumbar spine. TBS > 1.350 was considered indicative of normal microarchitecture, TBS between 1.350 and 1.200 indicated partially degraded microarchitecture, and TBS < 1.200 was considered degraded microarchitecture [24]. As in BMD, vertebrae with artifacts or fractures were excluded from the TBS measurements. The presence of vertebral fractures was determined, in the dorsal–lumbar tract of the spine, by morphometric X-ray absorptiometry scan [27], executed with the same densitometer and by the same skilled operators, using the quantitative method with the Vertebral Fracture Assessment (VFA).

### 2.4. Statistical Analysis

Statistical analysis was performed using Jamovi Version 2.3.28 [28]. Continuous variables were described as median and interquartile range (IQR), and the Mann–Whitney —test was applied. Qualitative data were expressed as frequencies and compared by χ2-test. A *p* < 0.05 was considered significant.

Taking into account the variables that could have a significant effect on bone microarchitecture, assessed by TBS, a uni- and multivariate analysis was performed in the total population, considering DM, BMI, RA, COPD, smoking habit, long-term cortisone therapy, and excessive alcohol intake; focusing only on the DM group, a uni- and multivariate analysis was performed considering DM duration, HbA1c levels, presence of chronic complications of DM, and specific hypoglycemic therapy. ANCOVA analysis was conducted to explore differences in the TBS value in BMI adjusted model considering DM. Odds ratio (OR) was calculated.

## 3. Results

Clinical records of 589 patients affected by fragility fractures were examined: median age was 80 ± 12 years, with 20 (3.3%) patients being males. The median BMI was 23.2 ± 3.33 kg/m^2^, and median age at the time of the first fracture was 71 ± 12 years. Median BMD in the lumbar spine was 0.763 ± 0.160 g/cm^2^, median BMD in the femoral neck was 0.572 ± 0.111 g/cm^2^, median BMD in the total hip was 0.700 ± 0.131 g/cm^2^, median TBS was 1.250 ± 0.114. A family history of osteoporosis was reported by 169 (28.3%) patients, smoking habit by 108 (18.1%), and alcohol excess by 6 (1%). A total of 21 (3.5%) patients were affected by RA, 35 (5.9%) patients by COPD, and 30 (5.0%) were on long-term cortisone therapy. Regarding the site of fracture, 500 (83.6%) patients presented vertebral fractures, 30 (5%) hip fractures, 23 (3.8%) a combination of vertebral and hip fractures, and 52 (8.7%) non-vertebral and non-hip fractures. A descriptive analysis is reported in Table 1.

In this population, 75 (12.7%) patients were affected by DM: specific characteristics are summarized in Table 2.

Bone and clinical characteristics of patients with DM were compared with those of the 523 patients without DM. The comparative analysis is shown in Table 1.

No significant differences were found in median age (*p* = 0.190) and median age at the time of the first fracture (*p* = 0.514). BMI and prevalence of males were significantly higher in DM group (respectively, *p* < 0.001 and *p* = 0.016).

Similarly, smoking habit was found to be higher in DM patients (*p* = 0.017). The presence of a family history of osteoporosis and long-term cortisone therapy was significantly higher in subjects without DM (respectively, *p* < 0.001 and *p* = 0.033). No significant differences were found in the prevalence of alcohol excess, RA, and COPD.

**Figure 1:**
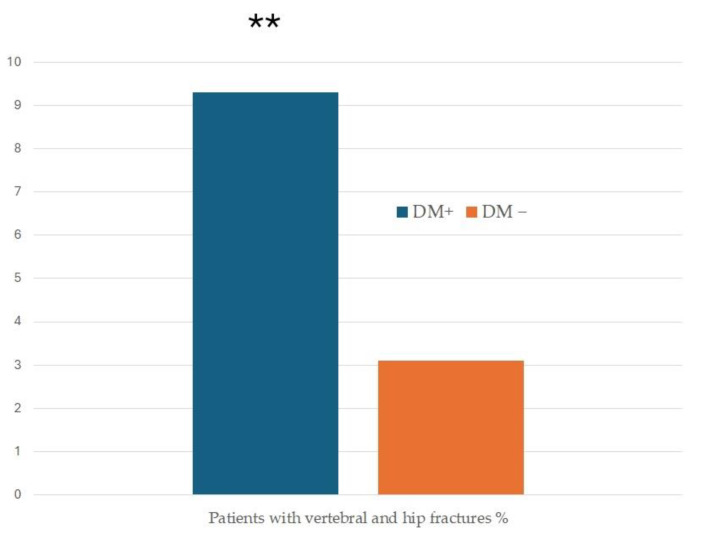
Prevalence of patients with a combination of vertebral and hip fracture. ** *p* = 0.008.

When analyzing bone characteristics, patients with DM showed a much more frequent combination of major fractures, such as vertebral and hip fractures (*p* = 0.008, OR 3.26 (CI 95% (1.30; 8.21)) (Figure 1).

**Figure 2:**
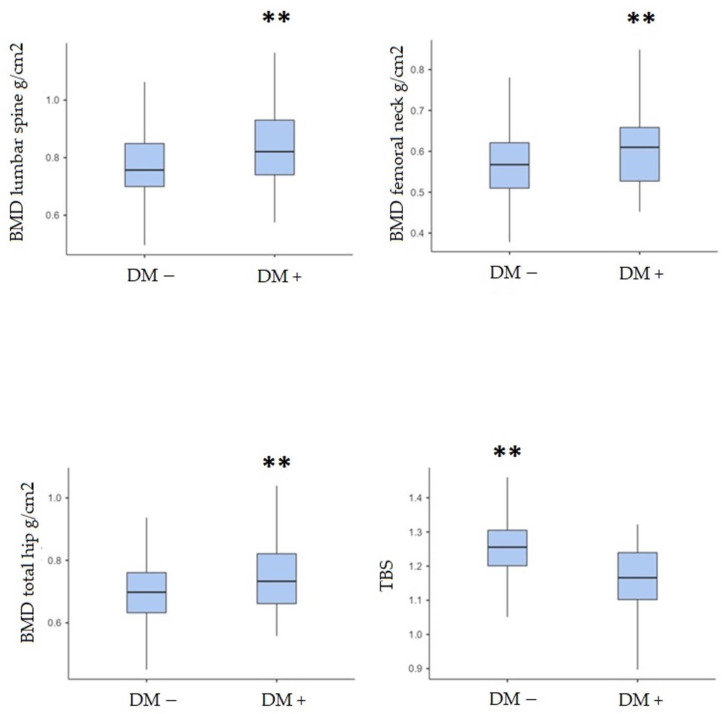
BMD and TBS values in DM patients and in control group (** *p* < 0.001; *p* = 0.006; *p* = 0.002; *p* < 0.001).

BMD values in DM patients were significantly higher at all the examined sites (lumbar spine *p* < 0.001; femoral neck *p* = 0.006; total hip *p* = 0.002). On the contrary, the median TBS was significantly lower in the DM population (*p* < 0.001) (Figure 2).

To investigate the presence of other variables that could have a significant effect on bone microarchitecture, assessed by TBS, a univariate analysis was performed: statistically significant results were found for BMI (t = −4.25; R^2^ = 0.0296; *p* < 0.001) and DM (t = −9.41; R^2^ = 0.130; *p* < 0.001). In the ANCOVA analysis, adjusted for BMI, a statistically significant difference was shown between the DM and the non-DM group in the TBS values (*p* < 0.001).

The multivariate analysis was conducted: only DM (*p* < 0.001) seemed to predict significantly reduced TBS value; presence of smoking habit, alcohol excess, RA, COPD, and long-term cortisone therapy did not show statistically significant results.

Both univariate and multivariate analysis including DM-related factors (DM duration, HbA1c values, presence of chronic complications of DM, specific hypoglycemic therapy) that may affect TBS value did not show a statistically significant effect.

## 4. Discussion

Several studies in the literature have highlighted a higher incidence of fragility fractures in patients with DM, compared to the general population [11,12,13,19,22,29], placing this disease among the causes of secondary osteoporosis [10]. Our analysis, carried out in a selected population of patients affected by fragility fractures due to severe osteoporosis belonging to a bone unit, revealed a prevalence of DM of only around 12%. Our results appear discordant with previous evidence from the literature. However, we cannot conclude that DM patients are less than expected in a group of patients with fragility fracture because our data are retrospectively collected from a Bone Unit database, where the presence of DM is recorded only according to an anamnestic information. We did not find statistical differences in median age at the time of the first fracture, so DM does not seem to cause an earlier development of bone fragility. A higher prevalence of males was found in the DM group: this result supports the evidence that males affected by DM may be exposed to a higher risk of developing bone fragility compared to those without DM [11,19]. Much more interesting data come from the bone structure analysis: the present research found that patients with DM have a higher association with combination of major fractures, such as vertebral and hip fractures, a circumstance that can lead to a worse clinical condition and more serious consequences for health.

This study found a notable prevalence of vertebral fractures in the population of patients who accessed the Bone Unit for the treatment of osteoporosis. This finding highlights the need to study patients with bone fragility in depth by prescribing not only DXA, but also morphometric examination, especially in the presence of suggestive signs and symptoms, such as spine pain or progressive height reduction.

As already reported in the literature, this study found higher levels of BMD in patients with DM and fragility fractures: this finding confirms that fractures tend to occur at higher BMD levels than in subjects without DM and that, beyond decreased BMD, bone fragility is sustained by numerous other factors that can influence bone quality [17,20]. It is important to focus attention on this specific characteristic because it may lead to a delay in the diagnosis and treatment of osteoporosis.

This phenomenon could be explained by the presence of insulin resistance and hyperinsulinemia, which determine an anabolic effect on tissues, including bone [16]; furthermore, the increased mechanical load on the bones caused by overweight and obesity [30], which often accompany DM, determines a positive stimulus for the deposition of bone mass. However, the new tissue shows compromised geometry and microarchitecture, especially due to hyperglycemia and AGEs that bind to collagen molecules, altering the normal molecular structure [8].

A similar diagnostic paradox occurs for bone fragility related to CKD: also in this case, especially in the more advanced stages of the disease, fragility fractures occur even in the absence of densitometric alterations [31]. CKD, as one of the most common chronic complications of DM, could contribute to the genesis of structural abnormalities of the bone. However, our research did not highlight significant negative correlations between TBS and the presence of diabetic nephropathy.

Due to the limited reliability of BMD in the diagnosis of osteoporosis in the DM population, the need for other diagnostic tools is emerging to improve the therapeutic path. Our study confirms that TBS, which evaluates the integrity of bone microarchitecture, is significantly reduced in patients with DM, even compared to other patients with compromised bone health [23,26]. However, it must be considered that TBS value can be influenced by numerous other factors, such as high BMI, smoking habit, excessive alcohol intake, chronic kidney disease (CKD), other chronic diseases, and the use of certain drugs [32]. Our research highlighted that the presence of DM and a higher BMI have a negative effect on the TBS value in the univariate analysis. In the multivariate analysis, only DM preserved a statistically significant influence. Smoking habit, excessive alcohol intake, RA, and long-term cortisone therapy do not appear to influence the TBS value in a significant way in the studied population. This result deserves particular attention; in fact, type 2 DM and high BMI are closely linked elements and affect a large part of the world population. A statistically significant difference in TBS value persisted between the DM and non-DM groups even in the BMI-adjusted analysis. As already highlighted in previous research, both DM and high BMI have negative effects on bone metabolism, however, showing non compromised densitometric values [33]. Previously, a high BMI and obesity were considered protective factors against bone loss and fractures. However, as for DM, obesity has been shown to represent a risk factor for the deterioration of bone health, being characterized by a proinflammatory state, adipose degeneration of the marrow, a greater incidence of other comorbidities, and a greater predisposition to motor deficits and falls [30]. The use of TBS as a diagnostic tool for DM-related bone fragility might be a valid aid in the clinical setting, aiming at early identification of high-risk subjects and prevention of the occurrence of negative outcomes. The appearance of fragility fractures presents heavy consequences: hip fractures, for example, have a mortality of 20% within the first year, leading to motor disability in more than 30% of cases. A total of 40% of patients experience a loss of autonomy and 80% experience a worsening of their quality of life [3]. In people affected by DM, glycemic control can worsen because of the stress related to trauma, surgery, and medications. Furthermore, healing times in presence of DM are prolonged, and the hospitalization and incidence of infectious complications after surgery is higher [34]. Immobility after a fracture, leading to a reduction in physical activity and mood, can cause a poor adherence to specific therapies, with a further increase in insulin resistance. The patient may be exposed to a greater risk of developing other chronic complications of DM, such as major cardiovascular events and pressure ulcers [35], which can also lead to death. Considering these risks, the fundamental need to make an early diagnosis of osteoporosis and prevent fragility fractures in this specific population must be highlighted. The main limitation of this study is its retrospective and monocentric nature.

## 5. Conclusions

According to our research, the prevalence of DM in the fragility fracture population does not appear to be particularly high, compared to the prevalence of the disease in the general older population. The development of fragility fractures is not anticipated in patients with DM, but they have a higher association with combinations of major fractures, such as vertebral and hip fractures. The instrumental analysis confirms that BMD tends to be higher at all sites, while TBS is significantly lower in DM individuals compared to non-DM ones, even when only patients with fragility fractures are taken into consideration. Therefore, our research, although with the limits described, suggests that TBS might be considered to improve diagnostic strategies and targeted assistance in this specific population. The importance of an early diagnosis and the prevention of bone fragility in patients with DM should be stressed and applied in everyday clinical practice, due to the heavy impact that fragility fractures may have on DM metabolic control and its progression towards chronic complications.

## Figures and Tables

**Table 1:** Descriptive and comparative analysis of patients divided according to presence of DM.

Variables	Total	DM+	DM−	*p*-Value
**Participants *n*. (%)**	589	75 (12.7)	523 (87.3)	
**Males *n*. (%)**	20 (3.3)	6 (8)	14 (2.7)	**0.016**
**Median Age (years)**	80 ± 12	82 ± 7.5	80 ± 12	0.190
**Median BMI (** **kg/m^2^** **)**	23.2 ± 3.33	24.6 ± 4.90	23.1 ± 3.27	**<0.001**
**Median age at the time of first fracture (years)**	71 ± 12	72 ± 13.5	71 ± 12	0.514
**Median BMD lumbar spine (** **g/cm^2^** **)**	0.763 ± 0.160	0.821 ± 0.190	0.757 ± 0.150	**<0.001**
**Median BMD femoral neck (** **g/cm^2^** **)**	0.572 ± 0.111	0.610 ± 0.131	0.567 ± 0.111	**0.006**
**Median BMD total hip (** **g/cm^2^** **)**	0.700 ± 0.131	0.733 ± 0.160	0.698 ± 0.129	**0.002**
**Median TBS**	1.250 ± 0.114	1.170 ± 0.137	1.260 ± 0.104	**<0.001**
**Family history of Osteoporosis *n*. (%)**	169 (28.3)	1 (1.3)	168 (32.1)	**<0.001**
**Smoking habit n. (%)**	108 (18.1)	21 (28)	87 (16.6)	**0.017**
**Alcohol excess n. (%)**	6 (1)	0 (0)	6 (1.1)	0.351
**RA *n*. (%)**	21 (3.5)	1 (1.3)	20 (3.8)	0.273
**COPD *n*. (%)**	35 (5.9)	4 (5.3)	31 (5.9)	0.838
**Cortisone therapy *n*. (%)**	30 (5.0)	0 (0)	30 (5.7)	**0.033**
**Vertebral fractures *n*. (%)**	500 (83.6)	57 (76.0)	443 (84.7)	0.057
**Hip fractures *n*. (%)**	30 (5)	5 (6.7)	25 (4.8)	0.484
**Both vertebral and hip fractures *n*. (%)**	23 (3.8)	7 (9.3)	16 (3.1)	**0.008**
**Non-vertebral—non-hip fractures n. (%)**	52 (8.7)	6 (8)	46 (8.8)	0.819

Qualitative data are expressed as absolute numbers (n.) and percentage of the total (%). Continuous variables are expressed as median and IQR. DM+: patients affected by diabetes mellitus, DM−: patients unaffected by diabetes mellitus. BMI: body mass index, BMD: bone mineral density, TBS: trabecular bone score, RA: rheumatoid arthritis, COPD: chronic obstructive pulmonary disease.

**Table 2:** DM patients’ specific characteristics.

Patients with DM n.	75
**Type 2 DM *n*. (%)**	68 (90.7)
**Median Duration of DM (years)**	15 ± 10
**Median HbA1c mmol/mol**	50 ± 12.3
**Median HbA1c %**	6.8 ± 1.5
**Nutritional therapy *n*. (%)**	10 (13.3)
**Oral hypoglycemic drugs *n*. (%)**	43 (57.3)
**Insulin *n*. (%)**	17 (22.7)
**Insulin + oral hypoglycemic drugs *n*. (%)**	5 (6.7)
**Coronary heart disease *n*. (%)**	15 (20)
**Carotid artery atheromasia—peripheral arterial disease *n*. (%)**	30 (40)
**Peripheral Neuropathy n. (%)**	15 (20)
**Retinopathy n. (%)**	8 (10.7)
**Nephropathy n. (%)**	16 (21.3)
**Stage 2 CKD n. (%)**	3 (19)
**Stage 3a CKD n. (%)**	5 (31)
**Stage 3b CKD n. (%)**	8 (50)

Qualitative data are expressed as absolute numbers (n.) and percentage of the total (%). Continuous variables are expressed as median and IQR. DM: diabetes mellitus, HbA1c: glycated hemoglobin; CKD: chronic kidney disease.

## Data Availability

The raw data supporting the conclusions of this article will be made available by the authors on request.
